# Supplementary material for: Revisiting the exposure criterion for PTSD: Using the COVID-19 pandemic as an opportunity to assess measurement invariance of PTSD symptoms across event types
Source: PLoS One. 2026 Apr 15;21(4):e0347315. doi: 10.1371/journal.pone.0347315 (PMC13082700; doi:10.1371/journal.pone.0347315)
Supplement: S1 Table — (DOCX) [file pone.0347315.s001.docx]

**S1 Table. Changes of the exposure criterion across subsequent versions of the DSM and ICD.**

| Version | Event definition | Exposure criteria |
| --- | --- | --- |
| DSM-III  (APA, 1980) | Recognizable stressor that would evoke significant symptoms of distress in almost everyone |  |
| DSM-III-R  (APA, 1987) | An event that is outside the range of usual human experience and that would be markedly distressing to almost anyone, involving serious threat to life or physical integrity | Direct exposure: Serious threat to one’s life or integrity.  Witnessed: Seeing another person who has recently been, or is being, seriously injured or killed as the result of an accident or physical violence.  Indirect exposure through others: Learning of exposure of one’s children, spouse, or other close relatives and friends.  Property destruction: Sudden destruction of one’s home or community. |
| DSM-IV  (APA, 1994) | A1. Traumatic event involving actual or threatened death or serious injury or a threat to physical integrity  A2. Intense fear, helplessness, or horror (in children, may be expressed by disorganized or agitated behavior | Direct exposure: Direct personal experience. Witnessed: Trauma to another person.  Indirect exposure through others: Learning of exposure to family member or other close associate/close friend |
| DSM-IV-TR | The person has been exposed to a traumatic event in which both of the following were present:? | The person experienced, witnessed, or was confronted with an event or events that involved actual or threatened death or serious injury, or a threat to the physical integrity of self or others.  The person's response involved intense fear, helplessness, or horror. Note: In children, this may be expressed instead by disorganized or agitated behavior. |
| DSM-5  (APA, 2013) | Exposure to actual or threatened death, serious injury, or sexual violence in one (or more) of the following ways (Age 6 years and older): | Directly experiencing the traumatic event(s).  Witnessing, in person, the event(s) as it occurred to others.  Learning that the traumatic event(s) occurred to a close family member or close friend. In cases of actual or threatened death of a family member or friend, the event(s) must have been violent or accidental.  Experiencing repeated or extreme exposure to aversive details of the traumatic event(s) (e.g., first responders collecting human remains; police officers repeatedly exposed to details of child abuse). Note: Criterion A4 does not apply to exposure through electronic media, television, movies, or pictures, unless this exposure is work related. |
| ICD-10  (WHO, 1993) | The patient must have been exposed to a stressful event or situation (either brief or long-lasting) of exceptionally threatening or catastrophic nature, which would be likely to cause pervasive distress in almost anyone. |  |
| ICD-11  (WHO, 2019) | Exposure to an event or situation (either short- or long-lasting) of an extremely threatening or horrific nature. | Direct exposure: experiencing natural or human-made disasters, combat, serious accidents, torture, sexual violence, terrorism, assault or acute life-threatening illness (e.g., a heart attack).  Witnessed: Witnessing the threatened or actual injury or death of others in a sudden, unexpected, or violent manner.  Indirect exposure through others: learning about the sudden, unexpected or violent death of a loved one. |
