# Supplementary material for: Revisiting the exposure criterion for PTSD: Using the COVID-19 pandemic as an opportunity to assess measurement invariance of PTSD symptoms across event types
Source: PLoS One. 2026 Apr 15;21(4):e0347315. doi: 10.1371/journal.pone.0347315 (PMC13082700; doi:10.1371/journal.pone.0347315)
Supplement: S2 Table — (DOCX) [file pone.0347315.s002.docx]

**S2 Table. Demographic information of the sample.**

|  | Total, n(%) | COVID-19, n(%) | Tradi-tional, n(%) | DSM-5 n(%) | Not DSM-5, n(%) | ICD-11, n(%) | Not ICD-11, n(%) |
| --- | --- | --- | --- | --- | --- | --- | --- |
|  | 72,851 (100.0%) | 35,639 (48.9%) | 37,212 (51.1%) | 6,674 (9.2%) | 66,177 (90.8%) | 33,858 (46.5%) | 38,993 (53.5%) |
| **Age in 7 classes** |  |  |  |  |  |  |  |
| 18-24 years | 3,773 (5.2%) | 1,779 (5.0%) | 1,994 (5.4%) | 543 (8.1%) | 3,230 (4.9%) | 1,882 (5.6%) | 1,891 (4.8%) |
| 25-39 years | 10,583 (14.5%) | 5,642 (15.8%) | 4,941 (13.3%) | 1,253 (18.8%) | 9,330 (14.1%) | 4,591 (13.6%) | 5,992 (15.4%) |
| 40-54 years | 14,668 (20.1%) | 7,438 (20.9%) | 7,230 (19.4%) | 1,596 (23.9%) | 13,072 (19.8%) | 6,447 (19.0%) | 8,221 (21.1%) |
| 55-64 years | 14,700 (20.2%) | 7,201 (20.2%) | 7,499 (20.2%) | 1,617 (24.2%) | 13,083 (19.8%) | 7,096 (21.0%) | 7,604 (19.5%) |
| 65-74 years | 18,246 (25.0%) | 8,583 (24.1%) | 9,663 (26.0%) | 1,095 (16.4%) | 17,151 (25.9%) | 8,333 (24.6%) | 9,913 (25.4%) |
| 75-84 years | 9,132 (12.5%) | 4,249 (11.9%) | 4,883 (13.1%) | 490 (7.3%) | 8,642 (13.1%) | 4,547 (13.4%) | 4,585 (11.8%) |
| 85 and older | 1,749 (2.4%) | 747 (2.1%) | 1,002 (2.7%) | 80 (1.2%) | 1,669 (2.5%) | 962 (2.8%) | 787 (2.0%) |
| **Gender in two categories** |  |  |  |  |  |  |  |
| Male | 29,388 (40.4%) | 14,814 (41.7%) | 14,574 (39.3%) | 2,716 (40.9%) | 26,672 (40.4%) | 13,444 (39.8%) | 15,944 (41.0%) |
| Female | 43,283 (59.6%) | 20,740 (58.3%) | 22,543 (60.7%) | 3,927 (59.1%) | 39,356 (59.6%) | 20,331 (60.2%) | 22,952 (59.0%) |
| **Urbanicity of residence** |  |  |  |  |  |  |  |
| (Very) strongly urban | 31,378 (43.1%) | 15,562 (43.7%) | 15,816 (42.5%) | 2,829 (42.4%) | 28,549 (43.1%) | 14,306 (42.3%) | 17,072 (43.8%) |
| Moderately to slightly urban | 35,050 (48.1%) | 17,036 (47.8%) | 18,014 (48.4%) | 3,282 (49.2%) | 31,768 (48.0%) | 16,521 (48.8%) | 18,529 (47.5%) |
| Non-urban | 6,423 (8.8%) | 3,041 (8.5%) | 3,382 (9.1%) | 563 (8.4%) | 5,860 (8.9%) | 3,031 (9.0%) | 3,392 (8.7%) |
| **Highest education level in 3 categories** |  |  |  |  |  |  |  |
| ISCED level ½ | 21,416 (29.5%) | 10,939 (30.8%) | 10,477 (28.3%) | 1,737 (26.1%) | 19,679 (29.8%) | 10,260 (30.4%) | 11,156 (28.7%) |
| ISCED level ¾ | 24,745 (34.1%) | 12,311 (34.7%) | 12,434 (33.6%) | 2,658 (40.0%) | 22,087 (33.5%) | 11,591 (34.4%) | 13,154 (33.8%) |
| ISCED level 5/6 | 26,423 (36.4%) | 12,275 (34.6%) | 14,148 (38.2%) | 2,253 (33.9%) | 24,170 (36.7%) | 11,857 (35.2%) | 14,566 (37.5%) |
| **Financial difficulties: difficulty to make ends meet** |  |  |  |  |  |  |  |
| No: no or little struggle | 59,310 (81.4%) | 28,396 (79.7%) | 30,914 (83.1%) | 5,018 (75.2%) | 54,292 (82.0%) | 27,690 (81.8%) | 31,620 (81.1%) |
| Yes: some or significant struggle | 13,296 (18.3%) | 7,146 (20.1%) | 6,150 (16.5%) | 1,629 (24.4%) | 11,667 (17.6%) | 6,030 (17.8%) | 7,266 (18.6%) |
| **Brief classification of country of birth** |  |  |  |  |  |  |  |
| Born in the Netherlands, both parents born in the Netherlands | 60,596 (83.2%) | 28,564 (80.1%) | 32,032 (86.1%) | 5,626 (84.3%) | 54,970 (83.1%) | 28,695 (84.8%) | 31,901 (81.8%) |
| Born in the Netherlands, parents born in Europe (excluding the Netherlands) | 2,188 (3.0%) | 1,038 (2.9%) | 1,150 (3.1%) | 180 (2.7%) | 2,008 (3.0%) | 1,033 (3.1%) | 1,155 (3.0%) |
| Born in the Netherlands, parents born outside Europe | 2,527 (3.5%) | 1,321 (3.7%) | 1,206 (3.2%) | 245 (3.7%) | 2,282 (3.4%) | 1,110 (3.3%) | 1,417 (3.6%) |
| Born in Europe,  excluding the Netherlands | 2,873 (3.9%) | 1,636 (4.6%) | 1,237 (3.3%) | 236 (3.5%) | 2,637 (4.0%) | 1,150 (3.4%) | 1,723 (4.4%) |
| Born outside Europe | 4,666 (6.4%) | 3,079 (8.6%) | 1,587 (4.3%) | 387 (5.8%) | 4,279 (6.5%) | 1,869 (5.5%) | 2,797 (7.2%) |
| **Time since the event** |  |  |  |  |  |  |  |
| 1 to 6 months ago | 14,445 (20.3%) | 4,153 (12.0%) | 10,292 (28,1%) | 1,061 (16.2%) | 13,384 (20.7%) | 7,451 (22.3%) | 6,994 (18.5%) |
| 6 to 12 months ago | 18,187 (25.5%) | 8,621 (25.0%) | 9,566 (26.1%) | 1,606 (24.5%) | 16,581 (25.7%) | 8,618 (25.8%) | 9,569 (25.3%) |
| More than 12 months ago | 38,562 (54.2%) | 21,767 (63.0%) | 16,795 (45.8%) | 3,888 (59.3%) | 34,674 (53.6%) | 17,341 (51.9%) | 21,221 (56.2%) |
