# Supplementary material for: Revisiting the exposure criterion for PTSD: Using the COVID-19 pandemic as an opportunity to assess measurement invariance of PTSD symptoms across event types
Source: PLoS One. 2026 Apr 15;21(4):e0347315. doi: 10.1371/journal.pone.0347315 (PMC13082700; doi:10.1371/journal.pone.0347315)
Supplement: S3 Table — (DOCX) [file pone.0347315.s003.docx]

**S3 Table. Checking for confounding factors in the differences in mean sum score across categories**

| **Total** | **Coef.** | **Intrusions** | **Coef.** | **Avoidance** | **Coef.** | **Negative alterations in cognition and mood** | **Coef.** | **Hyperarousal** | **Coef.** |
| --- | --- | --- | --- | --- | --- | --- | --- | --- | --- |
| Simple model: Criterion DSM | -3.811 | Simple model: Criterion DSM | -0.845 | Simple model: Criterion DSM | -0.381 | Simple model: Criterion DSM | -1.359 | Simple model: Criterion DSM | -1.226 |
| & time since exposure | -3.866 | & time since exposure | -0.857 | & time since exposure | -0.389 | & time since exposure | -1.372 | & time since exposure | -1.248 |
| & age | -3.166 | & age | -0.744 | & age | -0.305 | & age | -1.075 | & age | -1.042 |
| & gender | -3.726 | & gender | -0.825 | & gender | -0.373 | & gender | -1.323 | & gender | -1.204 |
| & urbanicity | -3.821 | & urbanicity | -0.847 | & urbanicity | -0.382 | & urbanicity | -1.363 | & urbanicity | -1.229 |
| & education | -3.827 | & education | -0.848 | & education | -0.383 | & education | -1.362 | & education | -1.233 |
| & financial difficulties | -3.163 | & financial difficulties | -0.691 | & financial difficulties | -0.315 | & financial difficulties | -1.124 | & financial difficulties | -1.033 |
| & migration background | -3.868 | & migration background | -0.862 | & migration background | -0.389 | & migration background | -1.384 | & migration background | -1.242 |
| Total | Coëfficient | Intrusions | Coëfficient | Avoidance | Coëfficient | Negative alterations in cognition and mood | Coëfficient | Hyperarousal | Coëfficient |
| Simple model: Criterion ICD | -1.106 | Simple model: Criterion ICD | -0.754 | Simple model: Criterion ICD | -0.195 | Simple model: Criterion ICD | -0.139 | Simple model: Criterion ICD | -0.018 |
| & time since exposure | -1.054 | & time since exposure | -0.735 | & time since exposure | -0.191 | & time since exposure | -0.122 | & time since exposure | -0.007 |
| & age | -1.226 | & age | -0.774 | & age | -0.210 | & age | -0.191 | & age | -0.052 |
| & gender | -1.066 | & gender | -0.739 | & gender | -0.192 | & gender | -0.128 | & gender | -0.007 |
| & urbanicity | -1.134 | & urbanicity | -0.761 | & urbanicity | -0.199 | & urbanicity | -0.149 | & urbanicity | -0.025 |
| & education | -1.076 | & education | -0.741 | & education | -0.193 | & education | -0.135 | & education | -0.007 |
| & financial difficulties | -1.189 | & financial difficulties | -0.772 | & financial difficulties | -0.204 | & financial difficulties | -0.169 | & financial difficulties | -0.044 |
| & migration background | -1.275 | & migration background | -0.796 | & migration background | -0.216 | & migration background | -0.198 | & migration background | -0.055 |
