# Supplementary material for: Revisiting the exposure criterion for PTSD: Using the COVID-19 pandemic as an opportunity to assess measurement invariance of PTSD symptoms across event types
Source: PLoS One. 2026 Apr 15;21(4):e0347315. doi: 10.1371/journal.pone.0347315 (PMC13082700; doi:10.1371/journal.pone.0347315)
Supplement: S4 Table — (DOCX) [file pone.0347315.s004.docx]

**S4 Table. Factor loadings.**

| Domains | Fulfills DSM-5 exposure criterion | | Fulfills ICD-10 exposure criterion | | | Type of event | | |
| --- | --- | --- | --- | --- | --- | --- | --- | --- |
|  | Yes | No | Yes | No | | Pandemic-related | Traditional | |
| Intrusion |  |  |  |  |  |  |  |  |
| Item 1 | 0.80 | 0.75 | 0.75 | | 0.75 | 0.76 | | 0.74 |
| Item 2 | 0.78 | 0.73 | 0.74 | | 0.74 | 0.76 | | 0.73 |
| Item 3 | 0.83 | 0.79 | 0.79 | | 0.81 | 0.81 | | 0.79 |
| Item 4 | 0.88 | 0.82 | 0.82 | | 0.84 | 0.84 | | 0.82 |
| Item 5 | 0.82 | 0.75 | 0.76 | | 0.77 | 0.77 | | 0.76 |
| Avoidance |  |  |  |  |  |  |  |  |
| Item 6 | 0.90 | 0.88 | 0.88 | | 0.88 | 0.89 | | 0.87 |
| Item 7 | 0.90 | 0.89 | 0.89 | | 0.89 | 0.89 | | 0.89 |
| Negative alterations in cognition and mood |  |  |  |  |  |  |  |  |
| Item 8 | 0.59 | 0.58 | 0.56 | 0.60 | | 0.62 | | 0.54 |
| Item 9 | 0.80 | 0.76 | 0.76 | 0.77 | | 0.78 | | 0.75 |
| Item 10 | 0.78 | 0.74 | 0.74 | 0.76 | | 0.77 | | 0.73 |
| Item 11 | 0.86 | 0.82 | 0.83 | 0.83 | | 0.84 | | 0.82 |
| Item 12 | 0.78 | 0.76 | 0.76 | 0.76 | | 0.77 | | 0.76 |
| Item 13 | 0.83 | 0.79 | 0.80 | 0.79 | | 0.80 | | 0.80 |
| Item 14 | 0.80 | 0.78 | 0.78 | 0.78 | | 0.79 | | 0.77 |
| Hyperarousal |  |  |  |  |  |  |  |  |
| Item 15 | 0.72 | 0.70 | 0.70 | | 0.70 | 0.72 | | 0.69 |
| Item 16 | 0.62 | 0.55 | 0.58 | | 0.55 | 0.58 | | 0.55 |
| Item 17 | 0.73 | 0.65 | 0.69 | | 0.65 | 0.65 | | 0.68 |
| Item 18 | 0.83 | 0.79 | 0.80 | | 0.80 | 0.80 | | 0.80 |
| Item 19 | 0.78 | 0.76 | 0.77 | | 0.77 | 0.77 | | 0.77 |
| Item 20 | 0.67 | 0.60 | 0.61 | | 0.61 | 0.61 | | 0.61 |
| Covariances |  |  |  |  |  |  |  |  |
| Intrusion, Avoidance | 0.85 | 0.77 | 0.76 | | 0.80 | 0.82 | | 0.76 |
| Intrusion, Negative alterations in cognition and mood | 0.82 | 0.76 | 0.77 | | 0.78 | 0.79 | | 0.76 |
| Intrusion, Hyperarousal | 0.83 | 0.75 | 0.76 | | 0.77 | 0.77 | | 0.76 |
| Avoidance, Negative alterations in cognition and mood | 0.78 | 0.73 | 0.72 | | 0.75 | 0.76 | | 0.72 |
| Avoidance, Hyperarousal | 0.75 | 0.68 | 0.68 | | 0.71 | 0.71 | | 0.68 |
| Hyperarousal, Negative alterations in cognition and mood | 0.91 | 0.89 | 0.90 | | 0.90 | 0.90 | | 0.90 |
