# Supplementary material for: Revisiting the exposure criterion for PTSD: Using the COVID-19 pandemic as an opportunity to assess measurement invariance of PTSD symptoms across event types
Source: PLoS One. 2026 Apr 15;21(4):e0347315. doi: 10.1371/journal.pone.0347315 (PMC13082700; doi:10.1371/journal.pone.0347315)
Supplement: S5 Table — (DOCX) [file pone.0347315.s005.docx]

**S5 Table. Multigroup SEM for COVID-19 specific events compared to non-COVID-19 events.**

| Model | ꭓ^2^ | p | CFI | TLI | RMSEA |
| --- | --- | --- | --- | --- | --- |
| Overall |  |  |  |  |  |
| Total | 29800.72* | 0.000* | 0.940* | 0.931* | 0.050* |
| COVID-19 related | 13826.13* | 0.000* | 0.944* | 0.935* | 0.048* |
| Traditional | 16124.10* | 0.000* | 0.936* | 0.926* | 0.051* |
| Multigroup (COVID-19 related versus traditional) |  |  |  |  |  |
| No constraints | 76603.26 | 0.000 | 0.917 | 0.904 | 0.079 |
| Same factor loadings | 77750.49 | 0.000 | 0.915 | 0.907 | 0.078 |
| Same factor loadings + intercepts | 83093.87 | 0.000 | 0.909 | 0.905 | 0.079 |
| Same factor loading + intercepts + error variances | 87712.66 | 0.000 | 0.904 | 0.905 | 0.079 |
