# Supplementary material for: Revisiting the exposure criterion for PTSD: Using the COVID-19 pandemic as an opportunity to assess measurement invariance of PTSD symptoms across event types
Source: PLoS One. 2026 Apr 15;21(4):e0347315. doi: 10.1371/journal.pone.0347315 (PMC13082700; doi:10.1371/journal.pone.0347315)
Supplement: S6 Table — (DOCX) [file pone.0347315.s006.docx]

**S6 Table. Multigroup SEM per age group.**

|  | DSM-5 | | | | ICD-11 | | | |
| --- | --- | --- | --- | --- | --- | --- | --- | --- |
| Age group: 18-24 |  |  |  |  |  |  |  |  |
| CFI | 0.917 | 0.915 | 0.912 | 0.908 | 0.907 | 0.906 | 0.905 | 0.902 |
| TLI | 0.905 | 0.908 | 0.908 | 0.909 | 0.893 | 0.897 | 0.901 | 0.903 |
| RMSEA | 0.081 | 0.079 | 0.079 | 0.079 | 0.085 | 0.084 | 0.082 | 0.081 |
| SRMR | 0.343 | 0.367 | 0.405 | 0.408 | 0.479 | 0.493 | 0.506 | 0.509 |
| Age group: 25-39 |  |  |  |  |  |  |  |  |
| CFI | 0.937 | 0.936 | 0.935 | 0.934 | 0.918 | 0.917 | 0.917 | 0.914 |
| TLI | 0.927 | 0.930 | 0.932 | 0.934 | 0.906 | 0.910 | 0.913 | 0.915 |
| RMSEA | 0.074 | 0.072 | 0.072 | 0.070 | 0.084 | 0.082 | 0.081 | 0.080 |
| SRMR | 0.367 | 0.376 | 0.442 | 0.445 | 0.391 | 0.412 | 0.425 | 0.426 |
| Age group: 40-54 |  |  |  |  |  |  |  |  |
| CFI | 0.911 | 0.934 | 0.934 | 0.932 | 0.916 | 0.915 | 0.914 | 0.911 |
| TLI | 0.912 | 0.925 | 0.928 | 0.929 | 0.904 | 0.907 | 0.910 | 0.912 |
| RMSEA | 0.079 | 0.073 | 0.072 | 0.071 | 0.083 | 0.082 | 0.080 | 0.079 |
| SRMR | 0.360 | 0.334 | 0.351 | 0.421 | 0.325 | 0.343 | 0.358 | 0.360 |
| Age group: 55-74 |  |  |  |  |  |  |  |  |
| CFI | 0.932 | 0.931 | 0.930 | 0.928 | 0.910 | 0.909 | 0.907 | 0.904 |
| TLI | 0.922 | 0.925 | 0.927 | 0.928 | 0.897 | 0.900 | 0.903 | 0.905 |
| RMSEA | 0.072 | 0.071 | 0.070 | 0.069 | 0.083 | 0.082 | 0.081 | 0.080 |
| SRMR | 0.309 | 0.319 | 0.392 | 0.040 | 0.299 | 0.311 | 0.336 | 0.336 |
| Age group: 75+ |  |  |  |  |  |  |  |  |
| CFI | 0.916 | 0.915 | 0.914 | 0.905 | 0.875 | 0.871 | 0.868 | 0.859 |
| TLI | 0.904 | 0.907 | 0.910 | 0.906 | 0.857 | 0.859 | 0.862 | 0.860 |
| RMSEA | 0.069 | 0.068 | 0.067 | 0.068 | 0.084 | 0.083 | 0.083 | 0.083 |
| SRMR | 0.300 | 0.328 | 0.427 | 0.440 | 0.273 | 0.289 | 0.312 | 0.315 |
