# Supplementary material for: Revisiting the exposure criterion for PTSD: Using the COVID-19 pandemic as an opportunity to assess measurement invariance of PTSD symptoms across event types
Source: PLoS One. 2026 Apr 15;21(4):e0347315. doi: 10.1371/journal.pone.0347315 (PMC13082700; doi:10.1371/journal.pone.0347315)
Supplement: S1 File — (DOCX) [file pone.0347315.s007.docx]

**S1 File. Questionnaire (translated to English)**

The following questions are about your experiences during the COVID-19 period. We kindly ask you to indicate whether you have experienced each event listed in column A since the beginning of the COVID-19 period. If "Yes," please mark in column B whether you are still affected by it.

|  | A: Have you experienced this event since the COVID-19 pandemic started? | | B: Are you still affected by the event now? | |
| --- | --- | --- | --- | --- |
|  | No | Yes | No | Yes |
| I personally experienced hospitalization due to COVID-19 | □ | □ | □ | □ |
| Someone significant to me was hospitalized due to COVID-19 | □ | □ | □ | □ |
| Someone significant to me passed away due to COVID-19 | □ | □ | □ | □ |
| Someone significant to me has experienced severe illness due to something else than COVID-19 | □ | □ | □ | □ |
| Someone significant to me passed away due to something else than COVID-19 | □ | □ | □ | □ |
| I was afraid that I or someone close to me would get infected with COVID-19 | □ | □ | □ | □ |
| In my job I have seen a lot of people be severely ill or pass away due to COVID-19 | □ | □ | □ | □ |
| Due to the COVID-19 social distancing measures, I could not say goodbye to someone significant to me | □ | □ | □ | □ |
| I experienced threats and/or physical violence | □ | □ | □ | □ |
| I experienced sexual violence | □ | □ | □ | □ |
| I experienced a life threatening accident (for example in traffic or during your job) | □ | □ | □ | □ |

Which of the events mentioned in the previous question was the most intense for you?

| □ | That I personally experienced hospitalization due to COVID-19 |
| --- | --- |
| □ | That someone significant to me was hospitalized due to COVID-19 |
| □ | That someone significant to me passed away due to COVID-19 |
| □ | That someone significant to me has experienced severe illness due to something else than COVID-19 |
| □ | That someone significant to me passed away due to something else than COVID-19 |
| □ | That I was afraid that I or someone close to me would get infected with COVID-19 |
| □ | That I have seen a lot of people be severely ill or pass away due to COVID-19 in my job |
| □ | That due to the COVID-19 social distancing measures, I could not say goodbye to someone significant to me |
| □ | That I experienced threats and/or physical violence |
| □ | That I experienced sexual violence |
| □ | That I experienced a life threatening accident (for example in traffic or during your job) |

When did this event occur? We mean the most intense event you have experienced since the start of the COVID-19 pandemic.

| □ | Less than 1 month ago |
| --- | --- |
| □ | Between 1-6 months ago |
| □ | Between 6-12 months ago |
| □ | More than 12 months ago |

Below is a list of issues that people sometimes experience after a very stressful event. Please read each description carefully while thinking about the event that was most intense for you. Indicate to what extent you have experienced each problem in the past 4 weeks.

|  | Not at all | A little bit | Moderately | Quite a bit | Extremely |
| --- | --- | --- | --- | --- | --- |
| Repeated, disturbing, and unwanted memories of the stressful experience? | □ | □ | □ | □ | □ |
| Repeated, disturbing dreams of the stressful experience? | □ | □ | □ | □ | □ |
| Suddenly feeling or acting as if the stressful experience were actually happening again (as if you were actually back there reliving it)? | □ | □ | □ | □ | □ |
| Feeling very upset when something reminded you of the stressful experience? | □ | □ | □ | □ | □ |
| Having strong physical reactions when something reminded you of the stressful experience (for example, heart pounding, trouble breathing, sweating)? | □ | □ | □ | □ | □ |
| Avoiding memories, thoughts, or feelings related to the stressful experience? | □ | □ | □ | □ | □ |
| Avoiding external reminders of the stressful experience (for example, people, places, conversations, activities, objects, or situations)? | □ | □ | □ | □ | □ |
| Trouble remembering important parts of the stressful experience? | □ | □ | □ | □ | □ |
| Having strong negative beliefs about yourself, other people, or the world (for example, having thoughts such as: I am bad, there is something seriously wrong with me, no one can be trusted, the world is completely dangerous)? | □ | □ | □ | □ | □ |
| Blaming yourself or someone else for the stressful experience or what happened after it? | □ | □ | □ | □ | □ |
| Having strong negative feelings such as fear, horror, anger, guilt, or shame? | □ | □ | □ | □ | □ |
| Loss of interest in activities that you used to enjoy? | □ | □ | □ | □ | □ |
| Feeling distant or cut off from other people? | □ | □ | □ | □ | □ |
| Trouble experiencing positive feelings (for example, being unable to feel happiness or have loving feelings for people close to you)? | □ | □ | □ | □ | □ |
| Irritable behavior, angry outbursts, or acting aggressively? | □ | □ | □ | □ | □ |
| Taking too many risks or doing things that could cause you harm? | □ | □ | □ | □ | □ |
| Being “superalert” or watchful or on guard? | □ | □ | □ | □ | □ |
| Feeling jumpy or easily startled? | □ | □ | □ | □ | □ |
| Having difficulty concentrating? | □ | □ | □ | □ | □ |
| Trouble falling or staying asleep? | □ | □ | □ | □ | □ |
