# Supplementary material for: Revisiting the exposure criterion for PTSD: Using the COVID-19 pandemic as an opportunity to assess measurement invariance of PTSD symptoms across event types
Source: PLoS One. 2026 Apr 15;21(4):e0347315. doi: 10.1371/journal.pone.0347315 (PMC13082700; doi:10.1371/journal.pone.0347315)
Supplement: Attachment — Submitted filename: Rebuttal.docx [file pone.0347315.s009.docx]

We thank the reviewers for their thoughtful and constructive comments on our manuscript titled “Revisiting the exposure criterion for PTSD: Using the COVID-19 pandemic as an opportunity to assess measurement invariance of PTSD symptoms across event types”. We appreciate the time and effort spent to reviewing our work, and we have carefully revised the manuscript in response to all comments. Below, we provide detailed responses.

**Reviewer #1**

**Comment 1: A major limitation is that the PCL-5 is not a diagnostic test for PTSD, but rather a self-report assessment. It cannot be established, therefore, that the individuals included in the analysis were diagnosable with PTSD. Since PTSD describes a clinical entity, with criteria of duration and disturbance in functioning being essential components, the results could be interpreted as showing that the factor structure of the PCL-5 does not vary in the general population experiencing different stressful events.**

Indeed, our study relies on self-report data rather than clinical interviews. We have clarified in the manuscript that we are not reporting clinical diagnoses, but rather assessing PTSD symptomatology as captured by the PCL-5, which is a well-validated and widely used measure.

To our knowledge, there are no measurement invariance studies using clinical diagnoses of PTSD: the operationalization of PTSD symptom clusters largely operates in questionnaire-based research. Self-report measures like the PCL-5 provide a reliable way to capture symptom variation across populations.

We have emphasized in the revised manuscript (line 345-358) that while clinical interviews are valuable, our findings are relevant for understanding how the underlying symptom structure behaves across event types in population samples. Future research could extend this work using clinical samples. However, due to current diagnostic criteria, a clinical sample of individuals exposed to certain COVID-19-related events that do not fit the current exposure criteria would be infeasible.

**Comment 2: In addition, the finding that PCL-5 scores were significantly higher in every one of the four subscore domains of PTSD symptomatology for individuals experiencing events meeting the DSM-5 exposure criterion needs to be emphasized and could be used to argue for the dimensional nature of trauma- and stress-related disorders and reactions. In addition, due to the significantly higher scores for events meeting the DSM-5 and ICD-11 criteria, the authors need to acknowledge the opposite conclusion which is that expanding the exposure criteria could risk expanding the diagnostic entity of PTSD to include related, milder instances of stress- or trauma-related reactions.**

We agree that reporting higher PCL-5 scores for events meeting the DSM-5 exposure criterion is important. We have added prevalence by event category based on DSM-5 diagnostic criteria, showing the proportion of respondents meeting at least one intrusion (B) symptom, one avoidance (C) symptom, two negative alterations in cognition and mood (D) symptoms, and two alterations in arousal and reactivity (E) symptoms.

As expected, prevalence is generally lower for events not meeting the exposure criterion (see table 3 in the Results section). Nonetheless, a portion of respondents in this group still meets criteria for probable PTSD, indicating that while the average severity is lower, symptomatology can be clinically relevant for affected individuals. These results highlight the dimensional nature of trauma responses without implying that all reactions to events that do not fulfil the exposure criterion are milder stress reactions.

Additionally, our proposal to include events that demonstrate measurement invariance of PTSD symptoms does not suggest that these events are identical to those currently meeting the exposure criteria in terms of PTSD symptom sum scores or prevalence. As events currently recognized in the exposure criterion, such as war, sexual violence, and natural disasters, differ in their psychological impact and prevalence (Kessler, 2017), this cannot be used as an argument against including new events within the exposure criterion. This is also described in the manuscript.

We have discussed this more explicitly in the revised manuscript, acknowledging differences in average scores while reinforcing that the measurement structure remains consistent across event types.

**Comment 3: More importantly, the fact that the results come from a population sample, rather than a clinical sample, risks mixing normal stress and trauma-related reactions with clinical ones. In my opinion, this is the most important limitation of this study.
The authors need to note that the scores of their sample were very low when compared to clinical samples of individuals with PTSD. This, again, hints at the conceptual leap necessary to conclude that the findings in this study apply beyond normal stress and trauma-related reactions. For a reference on proposed PCL-5 cutoff values, see: Pettrich, A., Schellong, J., Dyer, A., Ehring, T., Knaevelsrud, C., Krüger-Gottschalk, A., Nesterko, Y., Schäfer, I., & Glaesmer, H. (2025). Beyond one-cutoff-fits-all: determining cutoff values for the PTSD checklist for DSM-5 (PCL-5). European Journal of Psychotraumatology, 16(1), 2514878.** [**https://doi.org/10.1080/20008066.2025.2514878**](https://doi.org/10.1080/20008066.2025.2514878)

We agree that scores in our population sample are lower than in clinical samples, which is consistent with expectations. A clinical sample, by definition, includes individuals seeking treatment and thus scoring higher.

At the same time, our analyses demonstrate that trauma-related symptomatology can reach DSM-5 symptom thresholds even in a general population. Importantly, the study design allows us to examine events that would not be included in clinical studies due to the strict exposure criteria (e.g., certain COVID-19-related events). This perspective offers valuable insight into the range of symptom expression, while avoiding circular reasoning about clinical versus non-clinical cases.

From a public health perspective, it is also important to consider the broader societal impact. While prevalence is generally lower among events that do not fulfil the DSM-5 or ICD-11 exposure criterion, these events often affect a larger portion of the population. As a result, the absolute number of individuals reporting probable PTSD based on the PCL-5 can still be substantial, highlighting the relevance of these findings for population-level mental health monitoring and intervention planning. As mentioned in comment 2, while low on average, a proportion of our sample did meet the criteria for probable PTSD confirming this point.

**Comment 4:
In general, if the results applied to clinically diagnosed cases of PTSD, they would call into question what the DSM-5-TR states as: “Indirect exposure through learning about an event (Criterion A3) is limited to events affecting close relatives or friends that were violent or accidental (i.e., death from natural causes does not qualify). Such events include murder, violent personal assault, combat, terrorist attack, sexual violence, suicide, and serious accident or injury.” Indeed, the DSM-5-TR excluding natural deaths of loved ones, which could still be sudden and “traumatic”, does seem arbitrary. However, the evidence presented here does not clearly show this for clinical cases, and I believe, therefore, that the main conclusion of this study is overstated and needs revision. The main findings, that “all four PTSD symptom clusters (intrusion, avoidance, negative alterations in cognition and mood, and hyperarousal) are present in all groups,” are interesting and need to be stated without confusing population-level self-reporting of symptoms with clinically distinct entities.**

We acknowledge that our findings do not directly apply to clinically diagnosed PTSD. We have revised the manuscript to clarify that we do not report on clinical diagnoses.

Our key finding, that all four PTSD symptom clusters are captured consistently across event types, suggests that the latent structure of PTSD symptoms is preserved even for events outside the strict DSM-5/ICD-11 exposure criterion. While we cannot generalize to clinical diagnoses, these results indicate that the strict separation of event types may not fully capture the dimensional nature of PTSD symptoms. Replication in clinical samples is added as a suggestion for future research.

**Comment 5: In addition to the overstated conclusions, which are not based on clinical samples, the manuscript includes errors of language and table numbering, which further weaken the quality of the work. The entire manuscript needs careful revision of table numbering and citing in the text. Table 3 needs to be labeled as Table 2, and it is not mentioned in the text and should be cited when discussing differences in scores between events meeting DSM-5 or ICD-11 criteria. Similarly, Table 5 needs to be relabeled as Table 4.
All abbreviations need to be explained first, such as PCL-5 in the abstract. The differences between events in total PCL-5 scores and in every sub score need to be mentioned in the abstract. Statistical measures for goodness of fit similarly need to be mentioned in the abstract, or at least the relevant p-values.
Type-editing is needed for the entire manuscript to ensure accurate grammar and typing errors. For example, in the abstract, the sentence “We found The results show measurement invariance between events during the pandemic that did and that did not comply with the DSM-5 or ICD-11 exposure criterion” needs to be revised.**

Thank you for pointing out these errors. All table numbering has been carefully reviewed and corrected. Abbreviations, including PCL-5, are now defined at first mention. We have also conducted thorough type-editing to address grammar and clarity throughout the manuscript.

**Reviewer #2**

**This is a very good manuscript, perhaps more could have been written about the history of PTSD as a diagnosis, and the difference between the lay public understanding of trauma and the clinical diagnostic understanding.**

We thank the reviewer for the suggestion to include a brief discussion of the history of PTSD and distinctions between public and clinical understandings of trauma. We have added relevant background in the introduction (lines 24-35) to provide context without overextending the manuscript.

**Reviewer #3**

**Comment 1:"n=72.851" means "n=72,851"? I think the latter is more appropriate.**

This was a language-related error, in Dutch we use the dots and comma’s the other way around compared to English. It has now been corrected to the proper English format.

**Comment 2: In the "Participants" section, the number of the participants in this study and relevant information should be given.**

The ‘participants’ section in the methods had a wrong heading. We changed it to ‘study design’. In the results section (3.1 Demographic characteristics), the number of the participants in this study and their demographic information is given.

**Comment 3: In Table 1, the “x” implies "yes", but readers might misunderstand it as "no", a tick might not cause such ambiguity.**

Good suggestion, we adapted it in the revised manuscript, see table 1.

**Comment 4: The implications and future study directions should not be discussed in the "Conclusions" section, which made the conclusion so long.**

We have now restructured the discussion section to reflect this suggestion in the revised manuscript.
