# Supplementary material for: Revisiting the exposure criterion for PTSD: Using the COVID-19 pandemic as an opportunity to assess measurement invariance of PTSD symptoms across event types
Source: PLoS One. 2026 Apr 15;21(4):e0347315. doi: 10.1371/journal.pone.0347315 (PMC13082700; doi:10.1371/journal.pone.0347315)
Supplement: Attachment — Submitted filename: Response to reviewer.docx [file pone.0347315.s010.docx]

**Response to Reviewer #1**

We thank Reviewer #1 for their feedback. We have revised the manuscript to address the points raised, in particular by softening the language to avoid overstating conclusions based on community-based, self-report data, clarifying the distinction from clinical diagnoses, and acknowledging alternative interpretations. Below, we detail the specific changes made.

**Comment:** The previous version used overly strong language, including claims such as “seriously question” and “individuals with PTSD experience the same disorder irrespective of the event,” which overextended the implications of our community-based, self-report study. Alternative interpretations, including dimensional approaches to trauma, should be acknowledged, and limitations regarding clinical generalization should be emphasized.

**Response:** We have revised the manuscript to ensure that the language is appropriately cautious and that limitations and alternative interpretations are explicitly acknowledged. Specific changes are as follows:

**1. Abstract**
**Original concern:** The abstract implied that the results directly challenge diagnostic manuals.
**Revision:** *These results suggest that the current exposure criteria in the DSM-5 and ICD-11 may not capture all relevant events and underscore the importance of further research to guide potential updates.*
**Rationale:** We softened the claim to “suggest” and emphasized that further research is needed, avoiding overstatement.

**2. Findings – Symptom clusters**
**Original concern:** The manuscript overstated that all individuals with PTSD experience the same disorder across events.
**Revision:** *First, all symptom clusters can be identified across the different types of events, which is consistent with the notion that PTSD symptoms reflect the same underlying latent structure irrespective of the precipitating event.*
**Rationale:** We now describe structural similarity of symptoms without claiming equivalence of clinical disorder.

**3. Findings – Exposure criterion**
**Original concern:** The manuscript implied the exposure criterion is flawed without acknowledging nuances.
**Revision:** *This study suggests that reliance on a narrow exposure criterion may be overly restrictive and that the exposure criterion may warrant reconsideration. Reassessing this criterion in light of a broader spectrum of potentially traumatic experiences may facilitate a more accurate understanding and identification of PTSD across diverse populations. The observed measurement invariance indicates that trauma-related reactions reflect similar underlying symptom clusters across event types, supporting the usefulness of a dimensional approach to trauma. At the same time, further research is needed to establish whether these findings generalize to clinically diagnosed samples.*
**Rationale:** We now clarify the implications, highlight the dimensional perspective, and emphasize the need for replication in clinical samples.

**4. Findings – COVID-19-related experiences**
**Revision:** *In addition, our findings support the view that COVID-19-related experiences can be meaningfully examined from a trauma perspective and that PTSD represents a relevant outcome in assessments of the pandemic’s mental health impact when symptom levels exceed established cut-off values.*
**Rationale:** We clarified that only symptom levels reaching clinically meaningful thresholds are relevant, addressing the concern about pathologizing normal stress reactions.

**5. Strengths & Limitations – Clinical generalization**
**Revision:** *Consequently, this study cannot draw conclusions about clinically confirmed PTSD diagnoses. At the same time, it is important to note that PTSD cannot be measured objectively [33]; both self-report and clinical interviews rely on subjective symptom reports. The PCL-5, while not a diagnostic tool, assesses the same underlying construct of PTSD symptoms and has demonstrated good correspondence with clinician-based diagnoses, including in Dutch validation studies [34]. While the current study has attempted to investigate whether PTSD symptoms arise following events that do not meet the formal exposure criterion using measurement invariance within the PCL-5, replication with clinician-administered diagnostic interviews is needed to determine whether the exposure criterion should be reconsidered in the context of clinically diagnosed PTSD.*
**Rationale:** This explicitly acknowledges the limitation of self-report data and the need for clinical validation.

**6. Implications – Avoiding pathologization**
**Revision: I***mportantly, our proposal to include events that demonstrate measurement invariance of PTSD symptoms does not imply that, similar to events that do meet the current stressor criteria, all events are equally severe. Only events associated with symptom levels reaching clinically meaningful thresholds, such as those captured by the PCL-5, are relevant in this context. In other words, the focus remains on trauma-related reactions that meet a clinically significant threshold, ensuring that normal stress responses in the community are not inappropriately labeled as PTSD.*
**Rationale:** This addresses the reviewer’s concern regarding potential overdiagnosis and demonstrates that the clinical relevance of symptoms has been considered.

**7. Conclusion – Exposure criteria**
**Revision:** *This study provides evidence of measurement invariance between events that meet the DSM-5 and ICD-11 exposure criteria and those that do not. These findings highlight that similar PTSD symptomatology can emerge following stressful events not currently recognized as qualifying exposures, raising questions about the scope of the current exposure criteria in both diagnostic manuals. Given this similarity, future research could explore whether extending diagnostic consideration to such events is warranted. In particular, the events assessed in this study may be relevant candidates for inclusion in the exposure criterion in clinical research settings, pending further validation.*
**Rationale:** Language has been softened, the claim is grounded in measurement invariance, and we explicitly note that clinical validation is necessary before altering diagnostic criteria.

**Summary:**
We believe these revisions address your concerns by:

- Softening strong claims regarding clinical implications
- Clearly distinguishing community-based, self-report findings from clinically diagnosed PTSD
- Explicitly acknowledging alternative interpretations (dimensionality, structural similarity)
- Highlighting the need for replication in clinically diagnosed samples
- Ensuring that the potential expansion of exposure criteria considers only individuals showing clinically meaningful symptom levels
