# Supplementary material for: Revisiting the exposure criterion for PTSD: Using the COVID-19 pandemic as an opportunity to assess measurement invariance of PTSD symptoms across event types
Source: PLoS One. 2026 Apr 15;21(4):e0347315. doi: 10.1371/journal.pone.0347315 (PMC13082700; doi:10.1371/journal.pone.0347315)
Supplement: Attachment — Submitted filename: Response to editor.docx [file pone.0347315.s011.docx]

Dear Editor

We are very glad to hear that the reviewers have now reviewed our revised manuscript and recommended publication.

- Regarding the suggestion to conduct a regression analysis examining the association between specific event types (Criterion A vs. non-Criterion A) and PTSD symptoms, we agree that this is an interesting question. However, it falls outside the aim of the current study. Our focus is not on identifying which events are more likely to be associated with PTSD symptoms, but on examining whether the distinction between Criterion A and non-Criterion A events is supported. The comparison between events with regards of sum score and prevalence rates are added as context. However, we already refer to studies that show that certain events (e.g., sexual violence and physical threats or violence) are more strongly associated with probable PTSD, and have added a reference to an article of our own using the same database that supports this finding: <https://doi.org/10.1016/j.jpsychires.2025.02.032>

- With regard to citing reviews on DSM-5 PTSD, ICD-11 PTSD, and complex PTSD prevalence, we have chosen not to include these. The aim of this study was not to estimate prevalence, and the prevalence rates observed in our data are inherently dependent on the specific selection and number of events included. Because of this, comparisons with broader prevalence reviews would be difficult to interpret and fall outside the scope of the current manuscript.

- We have revised the abstract to provide a more balanced summary of the study, not only the key findings about measurement invariance.

- Finally, we have explained in the methods how respondents who endorsed multiple events were handled. Participants were asked to select their most distressing (“worst”) event and to report their PTSD symptoms in relation to that specific event. This ensures that symptom reports are anchored to a single index event, limiting the influence of other events.

We hope these revisions address your suggestions. Finally, we want to thank you very much, besides the reviewers, for your time and the way you handled our manuscript and revisions.

Kind regards, also on behalf of my co-authors,

Anouk van Duinkerken
